# Topical delivery of meropenem via spanlastic carbopol gel: *in-vitro* studies and *in-vivo* application in pressure ulcers

**DOI:** 10.3389/fphar.2025.1672677

**Published:** 2025-09-25

**Authors:** Shadwa Abdelfattah, Fatma Mohamed Mady, Hatem A. Sarhan, Hazim O. Khalifa, Hamada Hashem, Hesham Hassan, Abdullah Alkhammash, Safy Hadiya, Reham Ali Ibrahem, Milad Reda Qelliny

**Affiliations:** ^1^ Department of Pharmaceutics, Faculty of Pharmacy, Minia University, Minia, Egypt; ^2^ Department of Pharmaceutics and Industrial Pharmacy, Faculty of Pharmacy, Merit University (MUE), Sohag, Egypt; ^3^ Department of Pharmaceutics, Faculty of Pharmacy, Minia National University, New Minia, Egypt; ^4^ Department of Veterinary Medicine, College of Food and Agriculture, United Arab Emirates University, Al Ain, United Arab Emirates; ^5^ Department of Pharmaceutical Chemistry, Faculty of Pharmacy, Sohag National University, Sohag, Egypt; ^6^ Department of Pathology, College of Medicine, King Khalid University, Asir, Saudi Arabia; ^7^ Department of Pharmacology, College of Pharmacy, Shaqra University, Shaqra, Saudi Arabia; ^8^ Assuit International Center of Nanomedicine, Al-Rajhy Liver Hospital, Assuit University, Asyut, Egypt; ^9^ Department of Microbiology and Immunology, Faculty of Pharmacy, Minia University, Minia, Egypt

**Keywords:** spanlastics, meropenem, transdermal, pressure ulcers, edge activator, mexA expression

## Abstract

**Introduction:**

Spanlastics, a type of elastic nanovesicle, represents a promising drug delivery system capable of encapsulating both hydrophilic and lipophilic drug compounds. These carriers are biodegradable, biocompatible, and non-immunogenic. Meropenem (MRP), a broad-spectrum carbapenem antibiotic, is widely used to treat severe infections in both adults and children before the causative pathogens are identified. However, meropenem’s aqueous formulations are highly unstable and must be administered within 24 h of preparation. This study aimed to develop a meropenem-loaded spanlastic formulation (MRP-SP) for topical application, aiming to enhance both the drug’s stability and skin permeability.

**Methods:**

Spanlastics were prepared using Span 60 and Brij 35 via the ethanol injection method. The MRP-SP formulation was extensively characterized through particle size analysis, polydispersity index (PDI), zeta potential, encapsulation efficiency, *in vitro* drug release, scanning electron microscopy, microbiological assays, and *in vivo* topical efficacy studies.

**Results and Discussion:**

The optimized formulation (Batch F5), composed of Span 60 and Brij 35 in a 1:4 M ratio, exhibited a particle size of 462 nm, spherical morphology, 69.5% drug encapsulation efficiency, and 20% drug release within 6 h. The gel form of the same batch showed a comparable release profile. Antibacterial testing revealed that MRP-SP reduced the minimum inhibitory concentration by 2.4-fold against Pseudomonas aeruginosa compared to free MRP. Additionally, MRP-SP significantly downregulated the expression of mexA, a key resistance gene. *In vivo*, the topical application of MRP-SP demonstrated superior therapeutic activity in treating ulcerative skin lesions in non-diabetic mice, as evidenced by wound closure percent (89% at 10 days), wound area (49% at 10 days), and histopathological improvements. Overall, the meropenem-loaded spanlastic formulation shows strong potential as an effective topical therapy for bacterial skin infections.

## Graphical Abstract

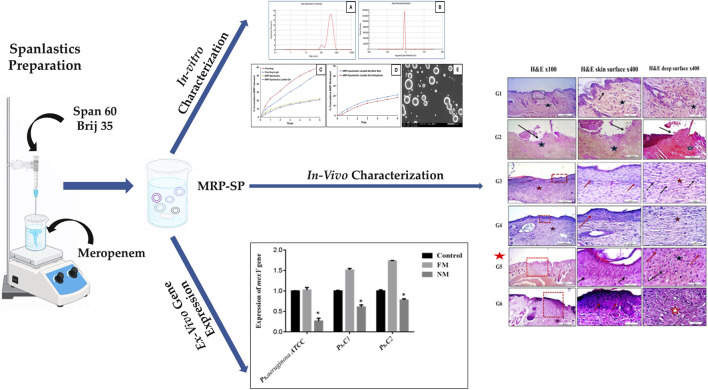

## 1 Introduction

Spanlastics, a subclass of nano-vesicular systems, have gained great attention and are particularly promising for delivering antimicrobial agents. These elastic vesicles are composed of a nonionic surfactant (e.g., Span 60) and an edge activator (e.g., Tween-80, Brij-35, or PEG400). Span 60-based spanlastics are more stable than those formed with Span 80, as their lipophilic nature supports the formation of lamellar structures (Kakkar and Kaur, 2011; Sahoo et al., 2014). The edge activators destabilize vesicle membranes, enhancing their deformability and drug delivery capacity. Spanlastics can be administered via multiple routes, including topical, oral, nasal, and transdermal, offering flexible delivery platforms for various therapeutic needs (Kakkar and Kaur, 2011). Spanlastics offer enhanced protection against drug degradation, exhibit superior skin permeability relative to niosomes and liposomes, enhance drug accumulation, and demonstrate favourable chemical stability, making them a suitable and efficient carrier system for antimicrobial drug delivery (Ansari et al., 2022; Wagdi et al., 2023).

Pressure ulcers (PUs), also referred to as pressure sores or bed sores or decubitus ulcers, are localized skin and tissue injuries that develop primarily over bony prominences due to prolonged pressure or pressure combined with shear forces (Langer et al., 2024). They remain a clinical challenge due to multifactorial etiologies, including friction, immobility, diabetes, infections, obesity, and vascular disorders. Despite the development of various animal models, an ideal preclinical model that closely mimics human pressure injury is still lacking, complicating research into pathogenesis and treatment (Kesarwani et al., 2021). Prevalence varies widely, ranging from 2.2% to 24.7% in hospitals and up to 34.1% in residential hospices. Risk is higher in older adults, immobile patients, those with diabetes, vascular disease, poor nutrition, or undergoing intensive care. Pressure ulcers negatively affect quality of life through pain, reduced independence, and limited social participation (Langer et al., 2024).

Pressure ulcer lesions are frequently colonized by either endogenous commensal microflora or exogenous environmental pathogens. The microorganisms implicated in PUs–associated infections are predominantly opportunistic, exhibiting pathogenic behavior under favorable conditions (Cereda et al., 2015; Gomes et al., 2022). Among the most commonly identified bacterial species are *Staphylococcus aureus*, *Proteus mirabilis*, *Pseudomonas aeruginosa* (investigated in the present study), and *Enterococcus faecalis*. Additional isolates reported in clinical studies include *Klebsiella pneumoniae*, *Staphylococcus epidermidis*, *Streptococcus viridans*, *Escherichia coli*, *Corynebacterium* species, *Bacteroides* species, as well as fungal organisms, particularly *Candida* species (Cereda et al., 2015; Gomes et al., 2022). Pressure ulcers are frequently complicated by opportunistic infections, and the increasing prevalence of antimicrobial resistance (AMR) among the associated pathogens further exacerbates treatment challenges and delays wound healing.

Antimicrobial resistance represents a major global public health concern, affecting both developed and developing countries (Dadgostar, 2019). It arises when bacteria or fungi develop resistance to existing antimicrobial agents, affecting individuals across all age groups and impacting human health, veterinary medicine, and agriculture (Ahmed et al., 2015; Khalifa et al., 2021). The reduced efficacy of antibiotics against infectious diseases threatens future treatment outcomes. AMR is associated with increased disease severity, extended hospital stays, elevated healthcare expenditures, greater reliance on costly second-line therapies, and higher rates of treatment failure (Dadgostar, 2019). In Europe, the economic burden linked to AMR exceeds nine billion euros annually. In the United States, the Center of Disease Control (CDC) reports an estimated 20 billion dollars in additional direct healthcare costs, alongside approximately 35 billion dollars in productivity losses each year (Dadgostar, 2019). These growing challenges highlight the urgent need for innovative drug delivery strategies to restore antibiotic effectiveness and overcome resistance mechanisms.

One innovative approach to combat AMR is the encapsulation of antibiotics in nanomaterials. Nanoparticle-based drug delivery systems can enhance antimicrobial bioavailability, reduce the likelihood of resistance development, and improve therapeutic outcomes (Aflakian et al., 2023). These nanoformulations can inhibit bacterial efflux pumps and penetrate biological barriers such as biofilms due to their small size, large surface area, and high reactivity (Blanco et al., 2015; Sharma et al., 2019; Tripathi and Goshisht, 2022; Khalifa and Al Khoori, 2025). Their ability to traverse microbial cell walls and biofilms, combined with favourable pharmacokinetics—such as prolonged plasma half-life and rapid renal clearance—further enhances their therapeutic potential (Blanco et al., 2015; Pelgrift and Friedman, 2013).

According to Fish (2006), Meropenem (MRP) is safe and effective for the treatment of complicated skin infections (Fish, 2006). Also, Elhardt, et al. (2023) stated that MRP could be used topically in eye for the treatment of infections (Elhardt et al., 2023). Briefly, MRP, a broad-spectrum carbapenem antibiotic, is widely used as an empirical treatment for severe bacterial infections when the causative organism is not yet identified. It is effective against a wide range of Gram-positive and Gram-negative pathogens and is indicated for conditions such as complicated intra-abdominal infections, bacterial meningitis, nosocomial pneumonia, skin and soft tissue infections, sepsis, febrile neutropenia, complicated UTIs, and gynecological infections. It remains a key therapeutic option, particularly for managing multidrug-resistant infections in hospitalized patients (Salmon-Rousseau et al., 2020).

At the end, PUs remain a significant clinical concern due to their complex pathophysiology, high prevalence, and frequent association with opportunistic infections. The rise of AMR further complicates treatment, limiting the effectiveness of standard antibiotics. Meropenem, a broad-spectrum agent, is effective against many pathogens found in infected ulcers but lacks established topical applications. Spanlastics offer enhanced drug stability, skin permeability, and localized delivery, while Carbopol gel improves retention and controlled release (Agha et al., 2023; Alaaeldin et al., 2021; Alharbi et al., 2022; Dhakane and Pandit, 2023). Developing a meropenem-loaded spanlastic gel provides a targeted strategy to improve therapeutic outcomes, reduce systemic exposure, and address infection-related delays in pressure ulcer healing.

## 2 Materials and methods

### 2.1 Materials

Meropenem (MRP) was obtained from Sigma Chemical Co. (St. Louis, MO, USA). Sorbitan monostearate (Span 60^®^), polyoxyethylene lauryl ether (Brij 35^®^), Carbopol 934, and polyethylene glycol 400 (PEG400) were also sourced from Sigma Chemical Co. Methanol, ethanol, citric acid, and sodium dihydrogen orthophosphate anhydrous were procured from ADWIC, El-Nasr Pharmaceutical Co. (Egypt). Sorbitan monooleate (Tween 80^®^) was purchased from Fluka Chemie GmbH (Germany). Dialysis tubing (cellophane membrane) with a molecular weight cut-off of 12–14 kDa was obtained from FREY Scientific (Northwest, USA).

### 2.2 Preparation of MRP-loaded spanlastic

Meropenem-loaded spanlastic formulations were prepared using the standard ethanol injection technique (Jaafar-Maalej et al., 2010). In this method, predetermined amounts of the nonionic surfactant Span 60^®^ and various edge activators (Tween 80, PEG400, and Brij 35) were dissolved in 3 mL of absolute ethanol at specified molar ratios. This organic phase was preheated to 55 °C to ensure complete dissolution of the components. Simultaneously, the aqueous phase—consisting of meropenem (MRP) dissolved in 10 mL of citrate-phosphate buffer (pH 5.5)—was maintained at 35 °C and stirred continuously at 500–600 RPM. The ethanol solution was slowly injected into the aqueous phase using a syringe fitted with a 24-gauge needle. Upon injection, ethanol was evaporated under continuous stirring, resulting in the spontaneous formation of spanlastic vesicles. To ensure complete ethanol removal, formulations were further processed and then sealed in aluminium foil and stored at 4 °C overnight to allow proper annealing of the lipid bilayer (El-Emam et al., 2025; Jaafar-Maalej et al., 2010). Blank spanlastic vesicles (without MRP) were prepared following the same protocol. All formulations were prepared in triplicate (n = 3).

### 2.3 *In-vitro* characterization of MRP-loaded spanlastic

The particle size (PS) and polydispersity index (PDI) of the spanlastic formulations were assessed using dynamic light scattering (DLS) via a Master-sizer 3000 E instrument (Malvern Instruments, UK). For analysis, each formulation was appropriately diluted by adding 10 μL of the spanlastic preparation to 980 μL of purified deionized water, and measurements were conducted at 25 °C (Torchilin and Weissig, 2003). Zeta potential was measured after diluting the spanlastics with Millipore water, using a mixture of 10 μL sample, 100 μL phosphate-buffered saline (PBS, pH 7.8), and 890 μL water, as per the protocol used for particle size analysis. To examine the surface morphology and structure of the spanlastic vesicles, scanning electron microscopy (SEM) was employed. A drop of the sample was placed on a glass stub, air-dried, and coated with a thin layer of gold using a sputter coater before SEM imaging (Ruozi et al., 2005).

The encapsulation efficiency (%EE) of meropenem within the spanlastic formulations was evaluated using both indirect and direct quantification methods. For the indirect method, 2 mL of each formulation was subjected to ultracentrifugation at 15,000×g for 2 h at 4 °C using a refrigerated centrifuge. This allowed the separation of free (unencapsulated) meropenem from the drug-loaded vesicles, facilitating accurate assessment of drug entrapment (Sharma et al., 2009). The concentration of free drug in the supernatant was then quantified by diluting it with a buffer solution (pH 5.3) and measuring the absorbance spectrophotometrically at 298 nm. The total drug content and encapsulation efficiency were subsequently calculated using standard equations (Khattab and Nattouf, 2021; López-Pinto et al., 2005).

$$EE\%=\frac{Total\;Amount\;of\;MRP-Unentrapped\;Amount\;of\;MRP}{Total\;Amount\;of\;MRP}\times 100$$

### 2.4 *In-vitro* and *ex-vivo* release profiles

*In vitro* release profiles of meropenem (MRP) from the prepared spanlastic formulations were evaluated using a modified Franz diffusion cell method with a dialysis membrane. Briefly, a specific volume of each MRP-loaded spanlastic formulation, containing an amount equivalent to 5 mg of MRP, was placed into a glass donor compartment sealed with a pre-soaked cellulose dialysis membrane (molecular weight cut-off: 12–14 kDa). The donor compartment was then immersed in citrate-phosphate buffer (pH 5.5), which served as the release medium to facilitate drug diffusion (Srinivas et al., 2010). The entire setup was maintained in a thermostatic shaker at 37 °C ± 0.5 °C and agitated at 50 ± 10 rpm. At predetermined time intervals over a 6-h period, 2 mL aliquots were withdrawn from the release medium and replaced with fresh pre-warmed buffer to maintain sink conditions. The concentration of MRP in the collected samples was measured spectrophotometrically at 298 nm. All experiments were conducted in triplicate, and the mean values were reported (Rajera et al., 2011).

*Ex vivo* permeation studies were performed to assess MRP diffusion across mouse skin using abdominal skin excised from female mice. Ethical guidelines were followed to minimize animal suffering and reduce the number of animals used. The abdominal hair was shaved, and after 24 h, the animals were euthanized via cervical dislocation. The skin was carefully excised, and subcutaneous fat was removed using a scalpel and isopropyl alcohol. The cleaned skin was then cut to appropriate dimensions, stored frozen, and used within 180 days. Prior to the permeation experiment, the frozen skin was thawed and soaked in 0.9% sodium chloride solution for 1 h. During the experiment, the skin was mounted between the donor and receptor compartments of the Franz cell, with the stratum corneum side facing the donor compartment and the dermal side facing the receptor medium. The permeation study was then carried out as previously described (Mostafa et al., 2018; Qiu et al., 2008).

### 2.5 Preparation and characterization of MRP-loaded carbopol 934 gel

A specific amount of Carbopol 934 was weighed to achieve a 1% (w/v) concentration and added to distilled water containing 0.01% (w/v) benzalkonium chloride, 3% (w/v) glycerol, and 1% (w/v) propylene glycol. The mixture was stirred for approximately 30 min using a homogenizer. To this, free MRP or MRP-loaded spanlastics, both at a concentration of 0.5% (w/v), were added. The mixture was further stirred for 20 min. The dispersion was allowed to hydrate for 60 min. A suitable amount of 1% NaOH was added to neutralize the Carbopol solution, and the mixture was stirred gently until a homogeneous gel was formed. The formulations were allowed to equilibrate at room temperature for 24 h (Mostafa et al., 2018). The gels were stored in amber-coloured glass vials and kept under sterilized conditions. The prepared gel formulations were evaluated by visual inspection, pH measurement, and rheological analysis. Viscosity measurements were performed using a Brookfield DV-III viscometer (Stoughton-MAO2072, USA), applying shear rates between 1 and 50 rpm. A plot of viscosity against shear rate was generated for each shear rate. All samples were maintained at 37 °C ± 1 °C during testing. After 1 minute of measurement, readings were recorded. The optimized MRP-loaded spanlastic formula (promising formulation) was subsequently evaluated for *ex-vivo* permeation using excised mouse skin (Table 1).

**TABLE 1:** Formulation of optimized MRP-loaded spanlastics carbopol 934 gel.

Formulation	Gelling agent	Gelling concentration (W/V%)	MRP concentration (W/V%)
Free Drug	Carbopol 934	1%	0.5%
MRP-Spanlastics			

### 2.6 Microbiological studies

#### 2.6.1 Antibiotic sensitivity of *Pseudomonas aeruginosa* to free and MRP-loaded spanlastics by the broth microdilution method

The antimicrobial activity of *Pseudomonas aeruginosa* ATCC 27853 as a reference strain and two other clinical strains (*Ps.C1* and *Ps.C2*) was evaluated using the minimal inhibitory concentration (MIC) method. Serial dilutions of both free and nanosized meropenem were prepared in a 96-well microdilution plate, with each well containing 0.01 mL of the drug in broth. Two-fold serial dilutions of free and nanosized meropenem were created. The inoculum was prepared by incubating *P. aeruginosa* isolates in Mueller-Hinton (MH) broth for 24 h. The bacterial cell density was adjusted to match the half McFarland opacity standard (108 CFU/mL) by diluting the culture in saline. The suspension was then diluted 1:100 in broth to achieve a final concentration of 106 CFU/mL. A 100 µL aliquot of this bacterial culture was added to each well containing 100 µL of the corresponding antimicrobial dilution in broth, resulting in a final bacterial concentration of 5 × 105 CFU/mL. The MIC was determined as the lowest concentration of the antibiotic that inhibited visible growth, as indicated by the absence of turbidity (Spencer et al., 2023).

#### 2.6.2 Assessment of efflux-pump (*mexA*) expression levels

##### 2.6.2.1 RNA extraction

All procedures were performed in an aseptic environment with controlled airflow to minimize RNase contamination. The centrifuge and vortex were placed inside the biological safety cabinet. To further reduce RNase contamination, all surfaces were wiped with 70% alcohol, and gloves were worn throughout the process.

##### 2.6.2.2 Buffers and reagents preparation

TE buffer was prepared by combining 30 mM Tris-HCl and 1 mM EDTA, and the pH was adjusted to 8. Fifteen milligrams of lysozyme were then added to 1 mL of the TE buffer.

##### 2.6.2.3 Cell-culture

Several colonies of *P. aeruginosa* ATCC 27853 (reference strain) and two clinical strains (*Ps. C1* and *Ps. C2*) were inoculated into LB broth and cultured in a shaking incubator. The cells were harvested once the optical density (OD) at 600 nm reached 0.5.

##### 2.6.2.4 Enzymatic lysis of bacterial cells and proteinase K digestion

All procedures were carried out at room temperature (25 °C). A 0.5 mL aliquot of each bacterial culture was centrifuged at 10,000 rpm for 10 min. The supernatant was discarded, and the resulting pellets were retained for further processing. Each pellet was resuspended in 200 μL of lysozyme-TE buffer and supplemented with 10 μL of proteinase K. The mixture was vortexed for 10 s to ensure proper mixing, then incubated at room temperature for 10 min in a shaking incubator. Subsequently, 700 μL of Buffer RLT and 7 μL of β-mercaptoethanol were added to each sample and vortexed vigorously. If visible debris remained, the samples were centrifuged again, and only the clear supernatant was used in the subsequent step. Finally, 0.5 mL of 100% ethanol was added to each tube and thoroughly mixed using a pipette.

##### 2.6.2.5 RNA purification

A volume of 700 µL of the prepared lysate was transferred into an RNeasy Mini spin column placed within a 2 mL collection tube. The tube was centrifuged at 10,000 rpm for 15 s, and the resulting flow-through was discarded. Next, 350 µL of RW1 buffer was added to the spin column, followed by centrifugation at 10,000 rpm for 15 s, after which the flow-through was again discarded. An additional 700 µL of RW1 buffer was added, and the column was incubated for 5 minutes before another spin at 10,000 rpm for 15 s. The spin column was then transferred to a clean Eppendorf tube. To wash the membrane, 500 µL of ethanol-containing RPE buffer was added, the column was tightly sealed and centrifuged at 10,000 rpm for 15 s. A second wash was performed by adding 500 µL of RPE buffer, sealing the column, and centrifuging at 12,000 rpm for 2 minutes. Subsequently, 30 µL of RNase-free water was added directly to the membrane in a fresh RNase-free collection tube, and the column was centrifuged at 12,000 rpm for 1 minute to elute the RNA. All RNA samples were stored at −80 °C until further use.

#### 2.6.3 cDNA synthesis

##### 2.6.3.1 cDNA reaction mixture contained

Complementary DNA (cDNA) synthesis was carried out using the T100 Gradient Thermal Cycler (Bio-Rad T100, USA). The reaction mixture was incubated at 42 °C for 10 min, followed by an additional incubation at 50 °C for 1 h. All procedures were performed in nuclease-free microtubes to avoid contamination. The experiment was conducted on ice, and all components were gently mixed by pipetting up and down. Initially, nuclease-free water, RNA sample, and primers were combined before the addition of other reagents. A positive RNA control was included to validate the experiment. The resulting cDNA samples were stored at −20 °C for future analysis (Table 2).

**TABLE 2:** Composition of cDNA reaction mixture.

Ingredient	Amount (µL)
RNase-free water	10.5
RNA template	2
dNTP (10 mM)	1
Script RT buffer (5X)	4
RNase inhibitor (40 units/μL)	0.5
DTT stock solution (100 mM)	1
Random primer	0.5
Script reverse transcriptase (200 units/μL)	0.5

#### 2.6.4 Assessment of efflux-pump (*mexA*)-expression levels among mutants

The relative expression of the *mexA* gene in *P. aeruginosa* strains was quantified using quantitative reverse transcription polymerase chain reaction (qRT-PCR). The primer sequences used (Table 3) were selected based on nucleotide data from GenBank (Shigemura et al., 2015). RNA extraction and synthesis of complementary DNA (cDNA) were conducted following previously described protocols (Liu et al., 2012). The qRT-PCR reactions were performed using a SYBR Green RT-PCR kit (Promega, USA) on a Bio-Rad CFX96 Touch Real-Time PCR Detection System (Bio-Rad, California, USA). The thermal cycling conditions included an initial denaturation at 95 °C for 5 min, followed by 40 cycles of denaturation at 95 °C for 30 s, annealing at 55 °C for 30 s, and extension at 72 °C for 30 s. A melting curve analysis was conducted with temperature increments of 0.5 °C every 5 s from 65 °C to 95 °C to verify product specificity. Gene expression levels were normalized to the ribosomal housekeeping gene *rpsL*. The relative expression of each target gene was calculated using *P. aeruginosa* ATCC 27853 as the calibrator strain (expression level set to 1). Each reaction was performed in triplicate, and results were expressed as the mean of three independent experiments (Tables 3 and 4).

**TABLE 3:** Primers used in the present study.

Primer	Sequence	References
mexA	F: AACCCGAACAACGAGCTG R: ATGGCCTTCTGCTTGACG	Quale et al. (2006)
rpsL	F: GCAACTATCAACCGACTGGTG R: GCTGTGCTCTTGCAGGTTGTG	Shigemura et al. (2015)

**TABLE 4:** Composition of qRT-PCR reaction mixture.

Component	Amount (µL)
Master-mix	10
Primer (F)-(10 pmol/μL)	1
Primer (R)-(10 pmol/μL)	1
Nuclease-free-water	6
Taq DNA polymerase	0.2
cDNA	2

### 2.7 *Ex-vivo* and *in-vivo studies*

#### 2.7.1 Animals

All animal procedures in this study were approved and licensed by the Animal Ethics Committee (AEC) of the Faculty of Pharmacy, Minia University (Protocol Code MPEC2401102). Ten-week-old male Wistar albino mice, weighing 20 ± 5 g, were obtained from the animal facility of the Faculty of Pharmacy, Assiut University. The mice were housed individually in standard cages under controlled environmental conditions: a 10:14-h light-dark cycle, temperature maintained at 23 °C–25 °C, and relative humidity between 65% and 85%. Food and water were provided *ad libitum* throughout the study (Kwek et al., 2021). The animals were randomly assigned into six groups, each comprising five mice (Table 5). Treatment protocols commenced upon the visible appearance of pressure ulcers, as assessed by gross observation twice daily for 14 days. Photographs documenting the gross morphology of wounds were taken every 2 days for all groups.

**TABLE 5:** Classification of animal experiments.

No.	Group	No. of rats	Class	Received
A. Untreated groups:				
1	Group (I)	5	Normal	Normal saline solution, 2 mL
2	Group (II)	5	Pressure ulcer	Normal saline solution, 2 mL
B. Control groups:				
3	Group (III)	5	Control	Blank Spanlastics in Carbopol 934 gel
4	Group (IV)	5	Control	Free drug in Carbopol 934 gel
C. Treated groups:				
5	Group (V)	5	Treated	Optimized spanlastics batch in Carbopol 934 gel
6	Group (VI)	5	Control	Fucidin^®^ ointment

#### 2.7.2 Microbiological studies

Swabs were collected from the intact skin of mice prior to any experimental procedures to assess the normal skin microbiota. Bed sore infection was induced using a well-characterized *P. aeruginosa* strain. A follow-up swab was taken 4 days post-infection to confirm successful colonization. A final swab was obtained at the end of the treatment period to evaluate the antibacterial efficacy of the applied formulations.

#### 2.7.3 Procedure of bed sore induction

To experimentally induce pressure ulcers (bedsores) in mice, a model based on ischemia-reperfusion injury was employed. A 2 × 2 cm area of dorsal skin was shaved, and a skin fold was gently elevated using artery forceps, then tightly secured with surgical thread to restrict blood flow and induce ischemia for two consecutive days. The ligature was then released for 1 day to allow reperfusion. This cycle of ischemia and reperfusion was repeated until the affected area exhibited visible signs of damage, progressing from redness to black discoloration and ulceration. The development of the ulcer was documented by photographing the area before treatment and every 2 days following the application of different therapeutic interventions (Kwek et al., 2021; Salcido Md et al., 2007).

#### 2.7.4 Anaesthetic procedure and skin preparation

The initial protocol utilized injectable anaesthetics. On Days 0 and 1, anaesthesia was induced in mice via intraperitoneal injection of ketamine hydrochloride (75 mg/kg). This approach was associated with prolonged recovery times; some animals required supplemental oxygen, and several needed reversals with Atipamezole hydrochloride (0.5 mg/kg, subcutaneously) to expedite recovery (Oh and Narver, 2024). Hair removal was performed by clipping the dorsal region after confirming adequate anaesthetic depth. Clippers were thoroughly cleaned before and after each use. Hair was clipped in the direction of growth, with the blade kept parallel to the skin surface. A second pass was made in the opposite direction to ensure complete hair removal. Remaining hair fragments were removed by gently wiping the area with sterile gauze moistened with sterile water or 0.9% saline solution.

#### 2.7.5 Treatment schedule

All treatment groups (G3, G4, G5, and G6) received their respective topical formulations at a defined dose (1 g), administered twice daily for 14 consecutive days (Lipsky et al., 2008). At the end of the treatment period, the animals were humanely euthanized by gentle cervical dislocation. To assess the histological effects of each gel formulation and ensure their safety for topical application, skin tissue samples were collected for histopathological evaluation. The samples were fixed, embedded in paraffin, and sectioned at a thickness of 5 μm. The sections were stained with hematoxylin and eosin (H&E) and examined under an Olympus light microscope (Japan) at a total magnification of ×400.

#### 2.7.6 Assessment of pressure ulcer management

##### 2.7.6.1 Microbiology in different stages

For bacterial culture, swabs were collected from mice prior to pressure ulcer induction, as well as from ulcer sites in five treatment groups on days 7, 10, and 14 following the initiation of treatment. These samples were used to assess the antimicrobial effectiveness of each intervention. Swabs were taken from a defined region where hair had been removed, using a sterile technique: the swab was gently rolled from the center of the area outward in a spiral motion, ensuring the boundaries were not exceeded. *Pseudomonas aeruginosa* was selectively isolated and identified using Cetrimide agar, a differential medium. Characteristic yellow green to blue pigmented colonies on Cetrimide agar indicated the presence of *P. aeruginosa*. The inoculated plates were incubated at 36 °C ± 1 °C for 18–24 h to allow for bacterial growth (Philips et al., 2015). At the same time, wound closure percentage, wound area, and wound closure time were recorded and measured at the predetermined days on 7, 10, and 14 following the initiation of the treatment using adjusted calibrator.

##### 2.7.6.2 Histopathological assessment of pressure ulcer

For histological evaluation of skin tissues, 2 × 2 cm sections were excised from the bed sore regions of both treated and untreated groups using a sterile scalpel and rinsed with normal saline. The collected samples were fixed in 4% formalin for 24 h. Following fixation, tissues were washed with phosphate-buffered saline (PBS) to eliminate residual formalin and then processed for paraffin embedding at the pathology laboratory of Assiut University Hospital. Thin paraffin sections (20 µm) were obtained using a microtome and stained with hematoxylin and eosin (H&E). The stained slides were examined under a light microscope, and representative images were captured for further evaluation and histopathological grading.

### 2.8 Statistical analysis

Statistical analysis was performed using Graphpad prism software version 7. Independent t-test and one-way ANOVA with *post hoc* tests were utilized for statistical analysis. The P value of <0.05 was statistically significant.

## 3 Results and discussion

### 3.1 Preparation of MRP-loaded spanlastics

To encapsulate MRP within an optimized spanlastic delivery system, the ethanol injection method was employed. Sorbitan monostearate (Span 60) was selected as the primary component for spanlastic preparation. This method is recognized for its simplicity, reproducibility, and reliability. The resulting spanlastic formulations were clear, uniform, and exhibited relative physical stability. Several formulation variables influence the final properties of spanlastics. Therefore, comprehensive preformulation and optimization studies were conducted to assess how these variables affect the physicochemical characteristics of the developed system (Supplementary Data). The key parameters examined in this study included MRP concentration, Span 60 M concentration, and the molar ratio between Span 60 and the selected edge activators (Abdelbari et al., 2021; Mazyed et al., 2021).

### 3.2 *In-vitro* characterization of MRP-Spanlastics

Following several optimizations and preformulation trials to determine the most suitable edge activator, molar ratio, and drug concentration, the optimized MRP-loaded spanlastic formulation was developed using Span 60 and Brij 35 as the edge activator at a 1:4 M ratio, with an MRP concentration of 5 mg/mL (Supplementary Data). The resulting formulation appeared clear and homogenous with a characteristic bluish opalescence, without any visible aggregates or phase separation. This bluish tint is attributed to the Tyndall effect; wherein light scattering occurs due to the presence of dispersed nanoparticles (Qelliny et al., 2019; Schaffazick et al., 2006).

The optimized spanlastic formulation exhibited a mean particle size of 462 nm and a polydispersity index (PDI) of 0.5, indicating an acceptable degree of size uniformity among the vesicles (Table 6). The mean vesicle size of ∼462 nm obtained in the present study is suitable for topical dermal delivery, where the therapeutic goal is in-skin retention and follicular targeting rather than transdermal flux, as in the treatment of bacterial infections in PUs. Previous studies have shown that vesicles in the 400–700 nm range penetrate deeply into hair follicles and form cutaneous depots, supporting our findings, while comparable spanlastic formulations reported for antipsoriatic, miconazole, AKBA, and eberconazole delivery further confirm the topical dermal applicability of this platform (Almutairy et al., 2024; Elmowafy et al., 2019; Haldankar et al., 2025; Ibrahim et al., 2023; Lila et al., 2025; Patzelt and Lademann, 2015; Patzelt et al., 2011). The zeta potential was measured at −7.4 mV, which reflects the influence of the surfactant and edge activator concentration on surface charge stability. It is important to know that in vesicular systems containing non-ionic surfactants—such as spanlastics—the dominant stabilization mechanism is steric rather than electrostatic. Non-ionic surfactants create a hydrated adsorbed layer around vesicles that prevents aggregation through osmotic and entropic repulsion when layers overlap (Ge et al., 2019; Tadros, 2013).

**TABLE 6:** *In-vitro* Characterization of optimized MRP-spanlastics.

Independent variables (studied factors)	Optimized values
Span: Edge Activator Ratio (mol%)	1:4 mol%
Drug Concentration (mg/mL)	5 mg/mL
Edge Activator Type	Brij 35

Responses	Obtained Results
Mean particle size (PS, nm)	462 nm
Polydispersity index (PDI)	0.5
Zeta potential (ZP, mV)	−7.4 mV
Encapsulation efficiency (EE, %)	69%
In-vitro drug release (%)	20% after 6 h
Surface Morphology (SEM)	Spherical

**FIGURE 1:**
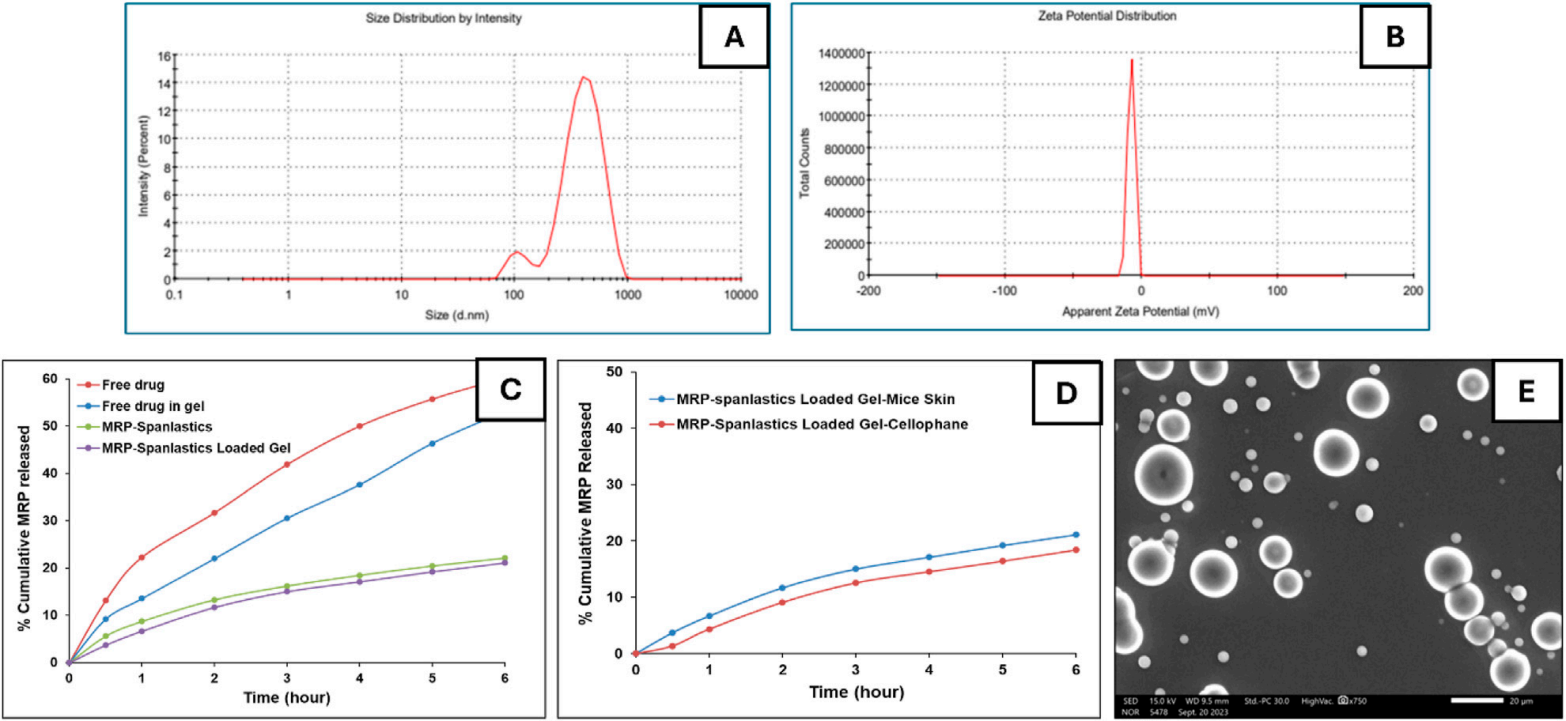
*In vitro* characterization of optimized MRP-spanlastics batch. The prepared MRP-Spanlastics batch exhibited mean particle size of 462 nm **(A)** Zeta potential of −7.4 mV **(B)** % cumulative MRP release of 20% after 6 h when compared to free MRP of 60% **(C)** *Ex-vivo* cumulative drug release showed minimal enhancement compared to *in vitro* drug release using cellophane membrane **(D)** and spherical spanlastics under scanning electron microscope **(E)**.

Regarding encapsulation efficiency, the formulation demonstrated a drug entrapment rate of 69%. Morphological analysis by scanning electron microscopy (SEM) revealed that the spanlastic vesicles were predominantly spherical in shape (Alwan and Abd Alhammid, 2023; Liu et al., 2023). SEM imaging also confirmed their three-dimensional structure (Figure 1). This morphology can be attributed to the amphiphilic nature of nonionic surfactants, which arrange themselves into bilayer vesicles in aqueous environments due to the opposing orientation of their hydrophilic and hydrophobic regions (Kazi et al., 2010). The spherical shape may also be energetically favorable, as it helps minimize surface free energy (Das and Palei, 2011). *In vitro* drug release studies (Figure 1) showed that the optimized MRP-spanlastic formulation released approximately 20% of the drug over 6 h, indicating a sustained release profile. In contrast, free MRP exhibited a rapid release of nearly 30% within the first 2 h, compared to only about 10% from the spanlastic formulation at the same time point (Figure 1).

### 3.3 Characterization of MRP-loaded spanlastics in carbopol 934 gel

The prepared gel formulation exhibited satisfactory clarity. The pH values of the MRP-loaded spanlastic formulation, plain Carbopol 934 gel, and the MRP-spanlastics incorporated into the Carbopol 934 gel are summarized in Table 7. According to recently published data, the optimal pH range for topical formulations designed for skin delivery lies between 4 and 6, which aligns well with the findings of the present study (Lukić et al., 2021). The gel demonstrated pseudoplastic (shear-thinning) behavior (data not shown), characterized by a reduction in viscosity with increasing shear rate. This shear-thinning property is advantageous for pharmaceutical gels, as it facilitates ease of formulation, spreading, handling, and topical application (Lin and Tsay, 2020).

**TABLE 7:** Characterization of MRP-loaded spanlastics in Carbopol gel.

Gel formulation	pH	Clarity	Rheology
Carbopol 934	3.1	Clear	Shear-thinning
Free MRP-Loaded Carbopol 934	4.6	Clear	Shear-thinning
MRP-Loaded Spanlastics in Carbopol 934	4.2	Clear	Shear-thinning

### 3.4 Skin permeation studies

To better understand the impact of gel formulation on the skin permeation of the optimized MRP-loaded spanlastics, an *ex-vivo* permeation study was conducted using Carbopol 934 gel and compared with spanlastics alone. Additionally, the permeation profile of free MRP incorporated into Carbopol gel was assessed. Owing to the absence of diffusion barriers, the free drug suspension exhibited a burst release, with approximately 22.5% of the loaded drug permeating within the first hour. After 6 h, the cumulative permeation reached 53% of the initial drug content (Figure 1). In contrast, encapsulating MRP within spanlastics significantly delayed its release into the receptor compartment. Furthermore, incorporating the spanlastics into a gel matrix introduced an additional diffusion barrier due to the gel’s network structure, further slowing drug permeation. Specifically, MRP-loaded Carbopol 934 gel allowed 3.7% and 21.11% of the drug to permeate at 1 and 6 h, respectively. These findings suggest that Carbopol 934 is a promising gelling agent for developing MRP-loaded spanlastic gel formulations, offering better control over drug release compared to other gelling agents (Data Not Shown).

### 3.5 Microbiological studies

#### 3.5.1 Antibiotic sensitivity of *Pseudomonas aeruginosa* to free and MRP-loaded spanlastics by broth microdilution method

*Pseudomonas aeruginosa* frequently colonizes PUs and can precipitate bacteremia, especially in hospitalized or long-term-care patients. In these chronic wounds, robust biofilm formation sustains inflammation and impedes healing. Its multidrug resistance reflects low outer-membrane permeability, inducible AmpC β-lactamase, upregulated RND efflux pumps (MexAB–OprM/MexXY), loss of OprD conferring carbapenem resistance, and target-site mutations, often yielding refractory infections (Elfadadny et al., 2024; Espejo et al., 2018; Lister Philip et al., 2009; Razdan et al., 2022). In the present study, the minimum inhibitory concentrations (MICs) of *P. aeruginosa* ATCC 27853 for free meropenem (FM) were determined to be 0.25 μg/mL. In contrast, the nano-formulated meropenem (NM) showed enhanced antibacterial activity, with an MIC of 0.06 µg/mL—indicating a fourfold reduction. For the clinical isolates, *P. aeruginosa* Ps.C1 and Ps.C2, the MICs for FM were 0.5 μg/mL and 1 μg/mL, respectively. Upon treatment with NM, the MIC values decreased to 0.125 μg/mL for Ps.C1 and 0.5 μg/mL for Ps.C2, reflecting a 2- to 4-fold reduction in resistance levels.

#### 3.5.2 Relative quantification of efflux pump expression

**FIGURE 2:**
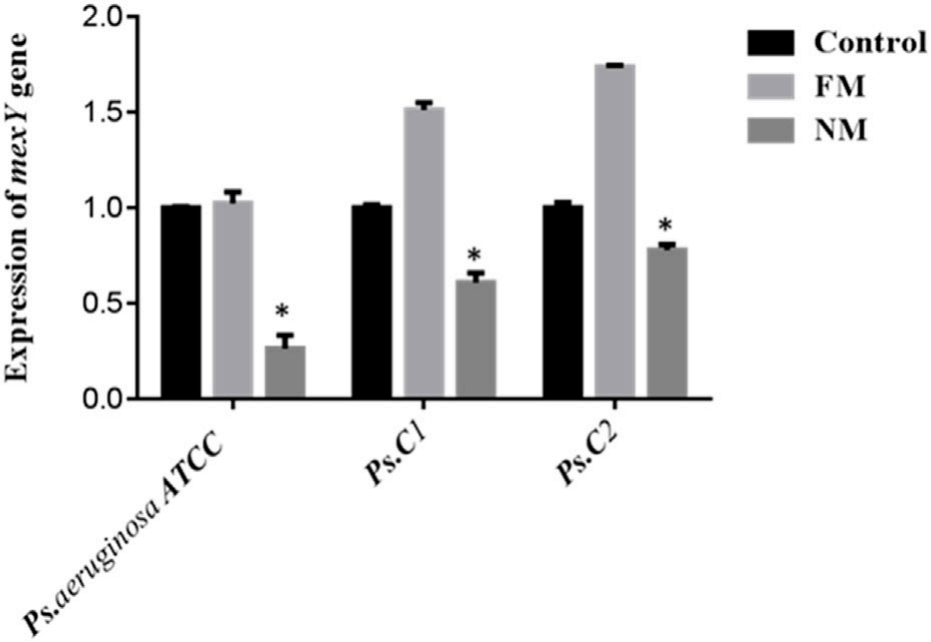
Expression of *mexA* in *Pseudomonas aeruginosa* ATCC strain (control strain) and two clinical strains (*Ps. C*1 and *Ps. C*2). FM (free meropenem treated strain), NM (Nanosized meropenem treated strain). **p-value <0.005, *p-value <0.05. The values are presented as mean ± SD (n = 3).

*mexA* encodes the membrane-fusion protein of the MexAB–OprM RND efflux pump in *P*. *aeruginosa,* a major driver of intrinsic multidrug resistance. *mexA* bridges the inner-membrane transporter MexB to the outer-membrane channel OprM, forming a trans-envelope conduit visualized by X-ray and cryo-EM. MexAB–OprM exports diverse antimicrobials—including fluoroquinolones, macrolides, tetracycline, chloramphenicol, novobiocin, and many β-lactams—while generally sparing imipenem; pump expression alters β-lactam susceptibility, particularly with β-lactamase derepression. mexAB-oprM transcription is normally repressed by MexR, NalC, and NalD; mutations or ligand-mediated derepression (novobiocin, pyocyanin) upregulate expression. Clinically, MexAB–OprM overproduction—often with MexXY—is frequent in multidrug-resistant and cystic fibrosis isolates, across diverse clinical settings (Aguilar-Rodea et al., 2022; Pesingi et al., 2019; Trépout et al., 2007; Tsutsumi et al., 2019). In the present study, the expression of the *mexA* gene was found to be upregulated in *P. aeruginosa* ATCC and two clinical isolates (Ps.C1 and Ps.C2) following exposure to free meropenem, compared to their respective untreated parental strains. In contrast, treatment with nano-formulated meropenem resulted in significantly reduced *mexA* expression levels of 1.263 in *P. aeruginosa* ATCC, 1.608 in Ps.C1, and 1.778 in Ps.C2—compared to the free meropenem group, which showed higher expression levels of 2.024, 2.512, and 2.736, respectively. The observed downregulation of *mexA* in the nano-meropenem-treated strains was statistically significant (*p* < 0.05), as illustrated in Figure 2.

**FIGURE 3:**
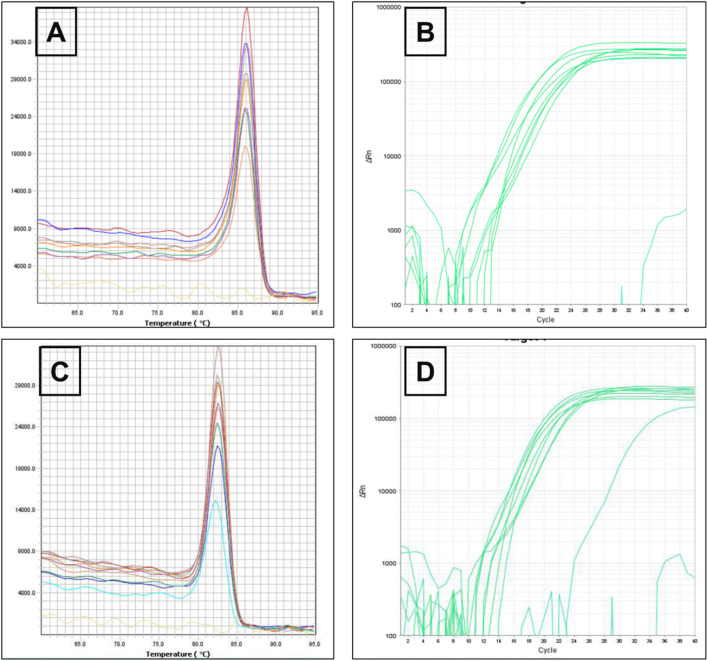
Melting curve of *rspL* gene **(A)** The amplification of *rspL* genes for *Pseudomonas aeruginosa* ATCC 27853 reference strains and *Pseudomonas aeruginosa* clinical strains **(B)** Melting curve of *mexA* gene **(C)** The amplification of *mexA* genes for *Pseudomonas aeruginosa* ATCC 27853 reference strains and *Pseudomonas aeruginosa* clinical strains **(D)**.

On the same way, *rpsL* gene encodes ribosomal protein S12 of the 30S subunit, a core component of the decoding center that ensures translational accuracy in bacteria (annotated in *P. aeruginosa* PAO1 as PA4260) (Alqarni et al., 2016; Meng et al., 2023). As illustrated in Figure 3, melting and amplification curves for both rspL gene and *mexA*. Panels A and C show single, sharp melt peaks (≈86 °C–87 °C for *rpsL*; ≈82 °C–84 °C for *mexA*), confirming specific amplicons without primer-dimers. Panels B and D display clean, sigmoidal amplification with early Cts, indicating robust detection of both targets across ATCC 27853 and clinical isolates. Because *mexA* (MexAB–OprM efflux) is present in all isolates, reductions in CFU and clinical burden after MRP-SP therapy imply the system overcame efflux capacity rather than eliminating the gene—plausibly via outer-membrane perturbation, ROS-mediated damage, or collapse of the proton-motive force that powers RND pumps.

### 3.6 *Ex-vivo* and *in-vivo* results

#### 3.6.1 Microbiology evaluation

**FIGURE 4:**
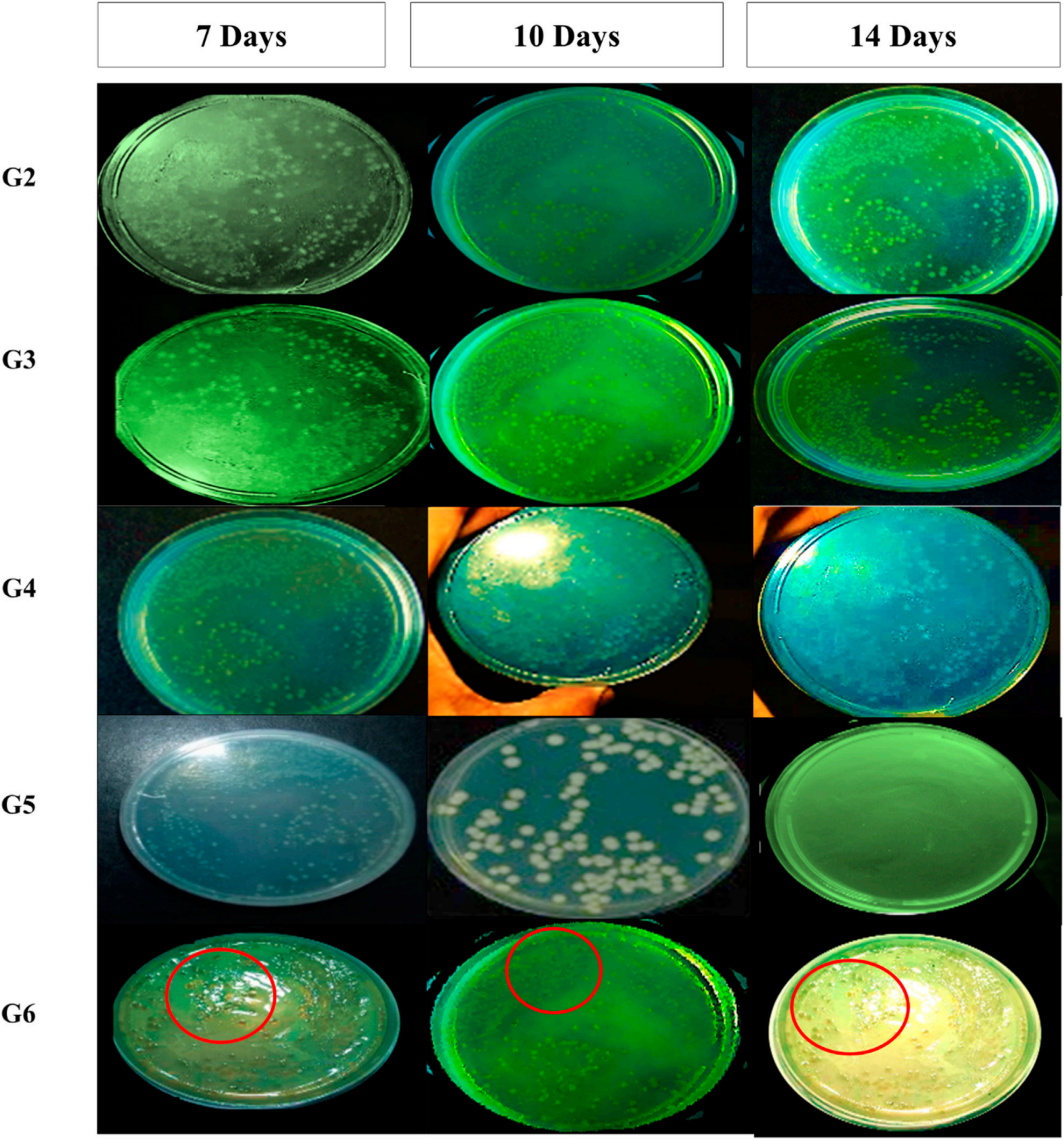
*Pseudomonas aeruginosa* colonies from wound swab of included mice on Cetrimide agar after treatment for 7-, 10-, and 14-days and incubation for 24 h at 37 °C. Colonies for group-6 (treated with marketed product) was labelled using red circles for more clearer visualization.

**FIGURE 5:**
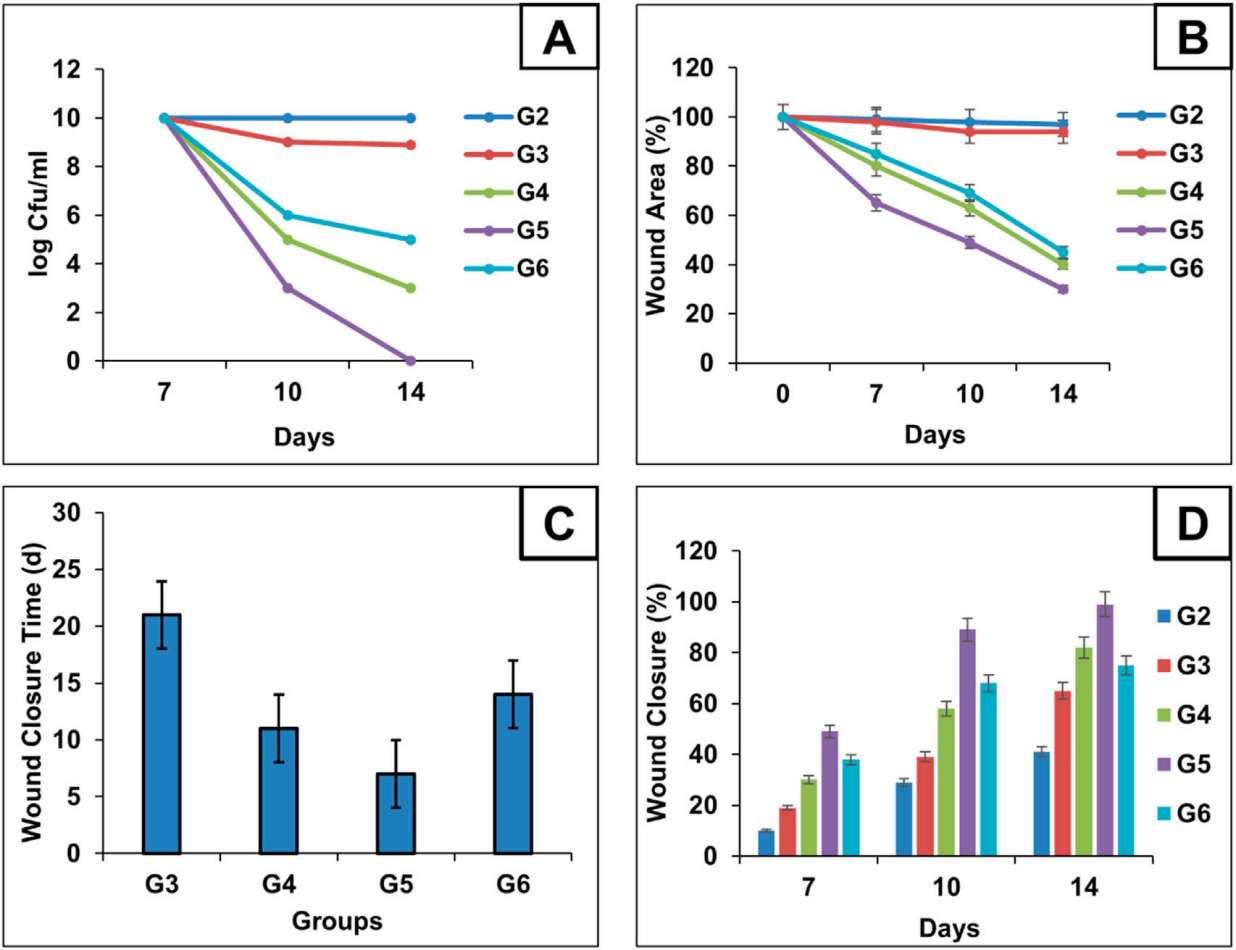
Microbial content for each group during treatment schedule were calculated using log CFU/mL and represented as Time-Kill analysis curves for each group **(A)** Chart illustrates the progression of wound epithelialization, represented as the percentage of residual wound area during treatment schedule **(B)** chart compares the total wound closure times across groups **(C)** and chart illustrates the percentage of wound healing at days 7, 10, and 14, reflecting the temporal healing dynamics among the treated mice **(D)**.

**FIGURE 6:**
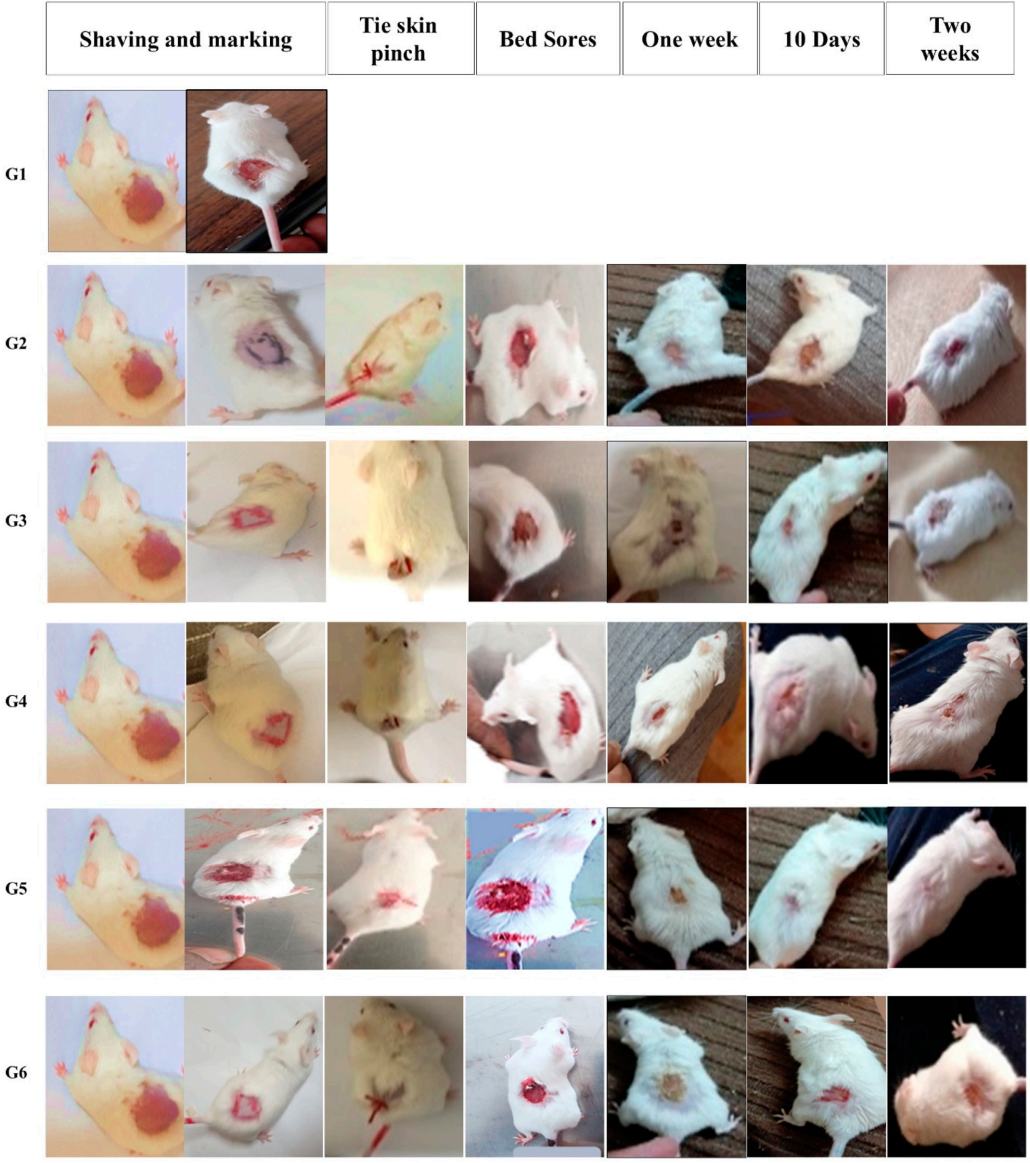
Gross photographic evaluation of wounds from different treatment groups at different intervals (days 7, 10, and 14). The figure illustrates the sequential experimental procedure, beginning with skin preparation steps including shaving and marking (column one). It then presents the induction of localized ischemia via skin ligation, establishing an ischemia–reperfusion model (column two, tie skin pinch). The progression of pressure-induced skin injury (column three, bed sore formation) is subsequently documented. The figure further delineates the designated treatment evaluation time points, specifically at 1 week (column four), day 10 (column five), and day 14 (column six), to monitor therapeutic response and wound healing process for each group.

Swabs were collected from infected mice before the initiation of treatment, as well as from ulcers in ten groups of mice—including five groups with pressure ulcers infected with *P. aeruginosa*—at 7-, 10-, and 14-days following treatment application. The time-kill curve analysis revealed that Fucidin required a significantly longer time to eliminate *P. aeruginosa* compared to both the optimized MRP-loaded spanlastic formulation in Carbopol 934 gel and the free MRP formulation in the same gel base (Figure 5A). Among the tested treatments, the MRP-loaded spanlastics demonstrated superior efficacy, achieving bacterial kill in a shorter duration than the free MRP gel formulation. The time-kill curve showed a gradual reduction in viable *P. aeruginosa* count over the first 10 days after exposure to the optimized spanlastic nanoparticles, followed by a sharp decline to undetectable levels (Figure 5A). These findings support the hypothesis that the outer membrane of *P. aeruginosa* exhibits limited permeability to hydrophilic drugs like free meropenem (MRP), serving as a key barrier to drug uptake (Figures 4–6).

On the same way, the antimicrobial effectiveness of MRP-spanlastics was evaluated using wound closure time, wound closure percent, and wound area percent (Figures 5B–D, respectively). As observed, MRP-spanlastics loaded Carbopol gel showed lowest wound area of 30% after 14 days compared to other groups, wound healing after 7 days, and approximately 89% wound closure percent after 10 days compared to 68% for marketed product (Figure 5). The enhanced antibacterial activity of the MRP-loaded spanlastics can be attributed to their improved permeability across the bacterial outer membrane, which occurs via a dual pathway—through porin channels and phospholipid bilayers. This enhanced penetration, combined with the direct interaction of the spanlastic nanoparticles with the bacterial membrane, increases membrane permeability and disrupts membrane integrity, thereby improving the antimicrobial efficacy of MRP (Aflakian et al., 2023).

#### 3.6.2 Histological (microscopic) evaluation

**FIGURE 7:**
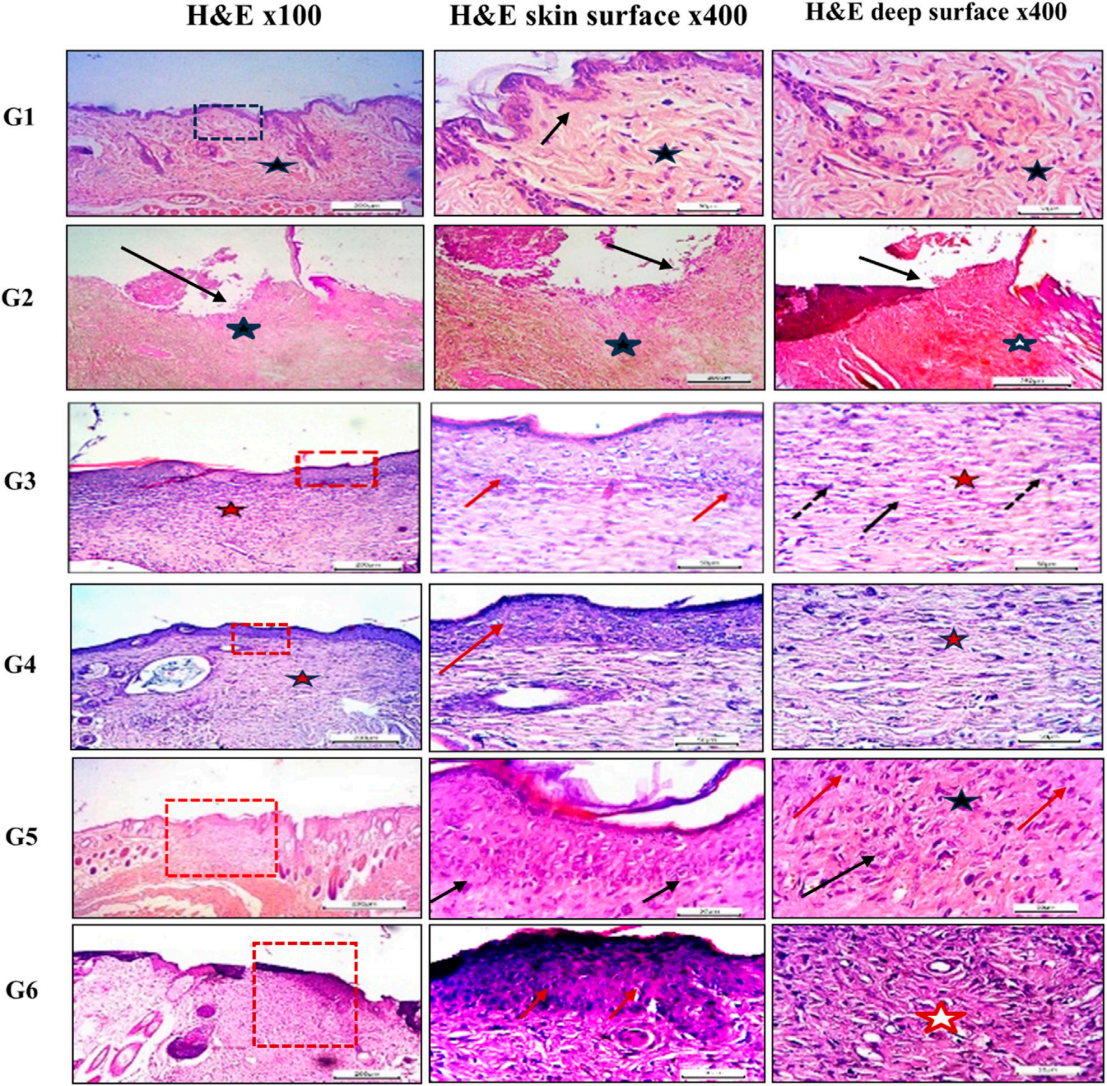
Histological sections of mice back skin from different treatment groups. Group 1 (normal skin): thin, uniform epidermis (dotted black square) with a distinct basal layer (black arrow) and mature collagen bundles in the dermis (black star). Group 2 (untreated bed sore): absence of epithelization (black arrow), hemorrhage (black star), and disorganized collagen (white star). Group 3 (Carbopol 934 gel): complete epithelization (dotted red square), hyperplastic epidermis (red arrows), regularly arranged collagen (red star), and mature fibrocytes (black arrows). Group 4 (Carbopol 934 gel + MRP drug): partial epithelization with poor adhesion (dotted red square), disorganized collagen (red arrow), and fibroblasts (red star). Group 5 (Carbopol 934 gel + MRP spanlastics): contracted healing with hyperplastic epithelial layer (dotted red square), regularly aligned collagen fibers (black arrows), and fibrocytes (black star). Group 6 (Fucidin ointment): healed ulcers with unhealthy epithelial cells (red dotted square), disorganized collagen (red arrows), and fibrosis (red star). Images captured at ×100 and ×400 magnification; scale bars = 200 μm and 50 μm, respectively.

Histopathological evaluation of hematoxylin and eosin (H&E)-stained paraffin-embedded skin sections under light microscopy revealed the following findings: The dorsal skin of normal, non-diabetic mice (Group 1, G1) displayed a typical architecture consistent with previous literature. The outermost layer (epidermis) comprised several layers of stratified squamous epithelium, covered by a thin keratinized layer. Beneath it, the dermis contained collagen bundles arranged in various orientations, interspersed with a few dark-stained nuclei of mature fibrocytes, and showed no evidence of inflammation (Figure 7). Hair follicles and associated sebaceous glands were also visible in the dermis. In contrast, untreated pressure ulcers (Group 2, G2 – negative controls) exhibited severe damage, with a detached or shredded epithelial surface, disrupted collagen structure, haemorrhagic areas, and notable infiltration of inflammatory cells. Mice treated with the plain spanlastic-loaded Carbopol gel (Group 3, G3) showed similar epithelial shredding and detachment, with the ulcer base containing disorganized collagen and infiltrating mononuclear inflammatory cells. Mice treated with free MRP-loaded Carbopol gel (Group 4, G4) demonstrated a regenerated but hyperplastic epithelial layer—thicker than normal. The ulcer bed displayed well-aligned collagen fibers, stained pink with eosin, and interspersed with dark blue, flattened nuclei of mature fibrocytes stained with hematoxylin. Remarkably, animals treated with the optimized MRP-loaded spanlastic formulation in Carbopol gel (Group 5, G5) showed significantly improved wound healing. The epithelial layer was intact and hyperplastic, yet healthy, with a well-defined basal layer. The underlying connective tissue exhibited organized, mature collagen bundles (marked with stars) and numerous dark-stained fibrocyte nuclei (indicated by dotted arrows). Conversely, the skin of mice treated with commercial Fucidin^®^ ointment (Group 6, G6) appeared hyperplastic but unhealthy, with poorly defined epithelial cells. Although collagen bundles were present in the dermis, they appeared disorganized and partially fibrotic (Figure 7).

## 4 Limitations and clinical relevance

The present study has some limitations that must be acknowledged. First, the *in-vivo* evaluations were restricted to non-diabetic murine models, which do not fully mimic the complex, multifactorial nature of pressure ulcers in human patients, particularly those with comorbidities such as diabetes, vascular insufficiency, or immobility. Secondly, the focus on *Pseudomonas aeruginosa* limits the generalizability against the polymicrobial nature of clinical pressure ulcers. However, the findings demonstrate strong clinical relevance by highlighting the potential of spanlastic-based meropenem formulations to enhance topical drug stability, permeability, and efficacy, offering a promising therapeutic strategy for resistant bacterial infections in chronic wounds.

## 5 Conclusion

MRP-loaded spanlastics were successfully formulated using the ethanol injection method, yielding promising outcomes. BRIJ 35 proved to be an effective edge activator, significantly enhancing meropenem (MRP) encapsulation efficiency. Key formulation variables—particularly the ratio of edge activator to Span 60—strongly influenced vesicle characteristics and drug entrapment capacity. Incorporation of the optimized spanlastics into Carbopol 934 gel resulted in a clear formulation with acceptable pH and favorable rheological properties, including pseudo-plastic behavior, while also exhibiting a more sustained drug release profile compared to the free drug gel. Antibacterial evaluation demonstrated a 2- to 4-fold reduction in the minimum inhibitory concentrations (MICs) against *P. aeruginosa*, likely due to the enhanced penetration capabilities of the nano-sized formulation and inhibition of bacterial efflux pumps. Histopathological analysis confirmed the superior wound healing potential of the optimized formulation (F5), especially in non-diabetic mice, indicating improved bioavailability and therapeutic effectiveness. Overall, MRP-loaded spanlastics represent a promising nanocarrier-based drug delivery platform for the treatment of bacterial infections and pressure ulcers.

## Data Availability

The original contributions presented in the study are included in the article/Supplementary Material, further inquiries can be directed to the corresponding author.
