# Supplementary material for: Topical delivery of meropenem via spanlastic carbopol gel: in-vitro studies and in-vivo application in pressure ulcers
Source: Front Pharmacol. 2025 Sep 25;16:1672677. doi: 10.3389/fphar.2025.1672677 (PMC12507946; doi:10.3389/fphar.2025.1672677)
Supplement: Supplementary file 1 [file Supplementaryfile1.docx]

Supplementary Material

# Physicochemical Characterization of Excipients Used in MRP-Spanlastics Preparation:

## Fourier-Transform Infrared

Trace (A) shows the FTIR spectrum of MRP, the stretching vibration peak of O-H in carboxylic acid group was observed at 2898.12 cm^−1^. The distinguishable peaks from the stretching vibrations of the C=O in the carboxyl group and β-lactam ring were observed at 1658.5 and 1756.8 cm^−1^, respectively. MRP are demonstrated at 3567.09 cm^−1^(OH stretching), 995cm ^-1^(C=C bending) and 3427.27 cm^-1^ (NH stretching). The stretching vibrations of the C-N bond in the pyrrolidine ring, β-lactam ring and dimethyl carbamoyl group were observed at 1189.45, 1253.5 and 1373.1 cm^−1^, respectively. In addition, the wagging and twisting vibrations of the C-H bonds in the hydroxyethyl substituent and the β-lactam ring were observed at around 1070 cm^−1^(Cielecka-Piontek et al. 2013). Trace (B) shows the characteristic FTIR peaks in the FTIR plots of Brij 35 of the broad absorption bands between 3259.26 cm^−1^ correspond to an OH group. peak -C=CH3 stretch at 2922.66 cm^-1^, carbonyl bands 1652.21 cm^-1^, and the strong Brij 35 absorption peak of a C-O stretch at 1102.81cm^-1^ in (CH2CH2O)_n_ (Maghsoodi et al. 2020). Trace (C) shows the FTIR spectrum of Span 60 such as aliphatic O-H stretching at 3330 cm^−1^, asymmetric and symmetric aliphatic C-H stretching at 2917.07 and 2849.58 cm^−1^, respectively, and C=O stretching of the ester at 1733.30 cm^-1^(Farmoudeh et al. 2020).

Trace (D) shows the FTIR spectrum of the physical mixture of span60 and MRP, such as aliphatic O-H stretching at 3330 cm^-1^, asymmetric and symmetric aliphatic C-H stretching at 2917.07 and 2849.58 cm^-1^, respectively, and C=O. Stretching of the ester at 1733.30 cm-1. MRP are demonstrated at 3567.09 cm^−1^ (OH stretching), 3427.27 cm^-1^ (NH stretching), 3013.62 cm^-1^, 2976.47 cm^−1^, 2934.86 cm^−1^, 2898.12 cm^−1^ (OH stretching in COOH), 1658.5 cm^−1^ (C-O stretching in COOH), 1189.45 cm^−1^ (CN stretching in pyrrolidine ring) and 668.47 cm^−1^ (OH bending in COOH). Trace (E) shows the FTIR spectrum of blank spanlastic formulation indicates characteristic bands of both Span 60 and Brij 35 However, the bands are less intense than the same bands in the individual components. The lipid bilayer formulation allows for the decreased intensity of the bands in the blank spanlastic formulations (Mangal et al. 2018). Trace (F) shows the FTIR spectrum of optimized MRP spanlastic, the peak of MRP at 3567.09 cm^−1^ disappeared, which might be attributed to potential hydrogen bonding. The encapsulation of MRP in the spanlastics was confirmed by the disappeared of N-H stretching (3427.27 cm^-1^), In addition to the multiple peaks of MRP that correspond to -OH stretching in COOH and peaks of C-O-H and O-H bending in COOH disappeared, which may be attributed to the ionic interactions. Also, the association of MRP in spanlastic was confirmed by the C=C bending (995cm^-1^). Furthermore, the peak of carbonyl band MRP were shifted to 1739 cm^-1^; which might be due to the electrostatic interactions.

## X-ray diffraction:

###### FTIR spectra of (A) MRP, (B) Brij35, (C) Span 60,(D) mixture, (E) blank spanlastic, (F) Optimized MRP loaded spanlastic formula.

######


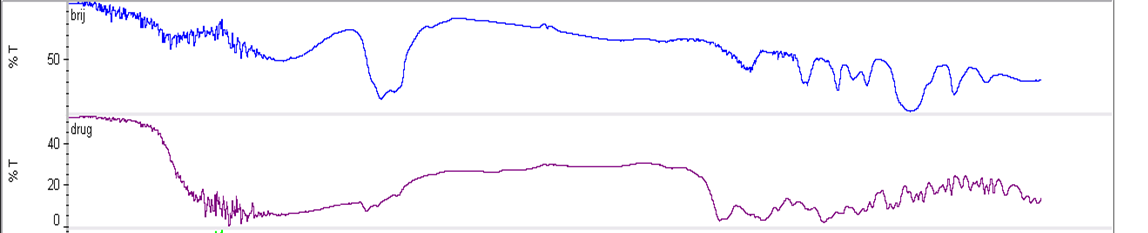


A


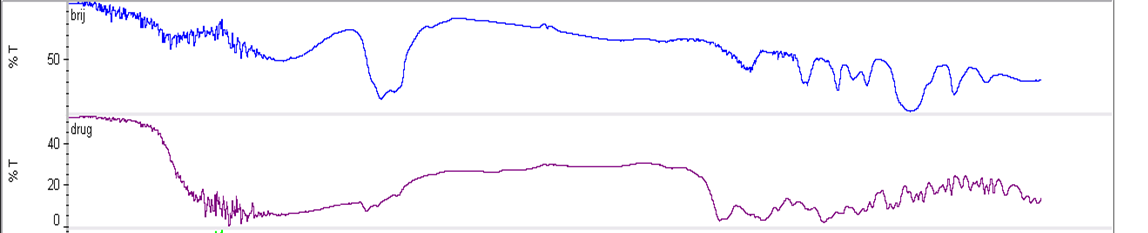


B


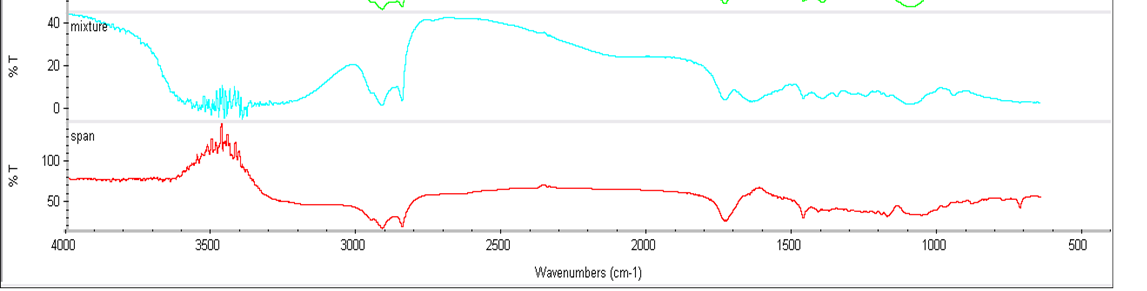


C


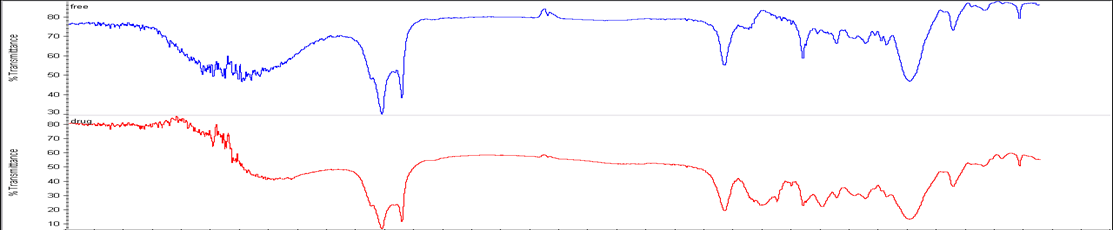


E

F


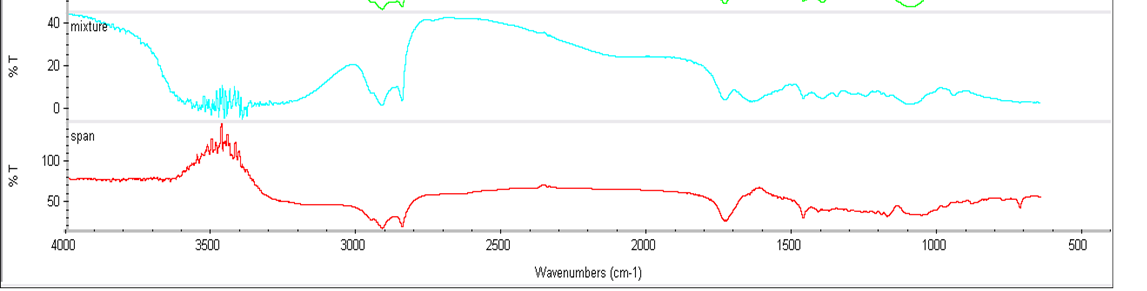


D


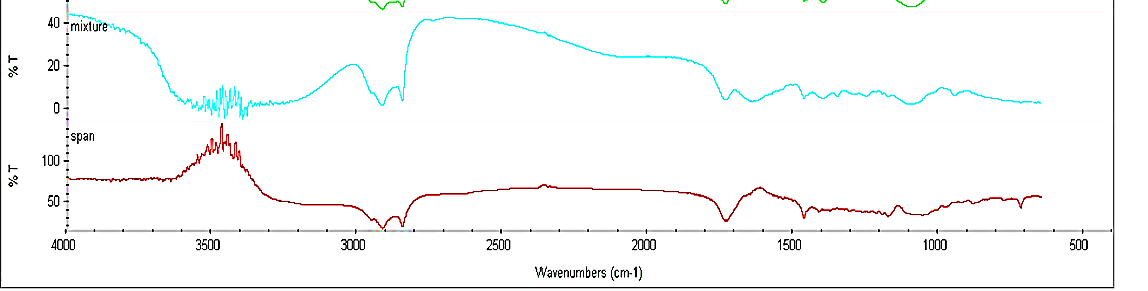


X-ray diffractograms of MRP Spanlastic XRD showed reduced intensity of 2θ scattered angles compared to pure MRP, indicating MRP's molecule distribution and lack of crystallinity. The X-ray diffractogram of the physical mixture showed no significant change in the intensity of MRP's 2θ scattered angles. This observation demonstrates that MRP was in a crystalline state in the formulation ingredients, ruling out the possibility of dilution causing the removal of MRP peaks in the Spanlastics (Mhango et al. 2017).

###### X-ray diffractograms of (A) pure MRP, (B) BRIJ35, (C) Span 60, (D) physical mixture of (E) blank spanlastics, (F) the optimized MRP loaded spanlastics formulation.


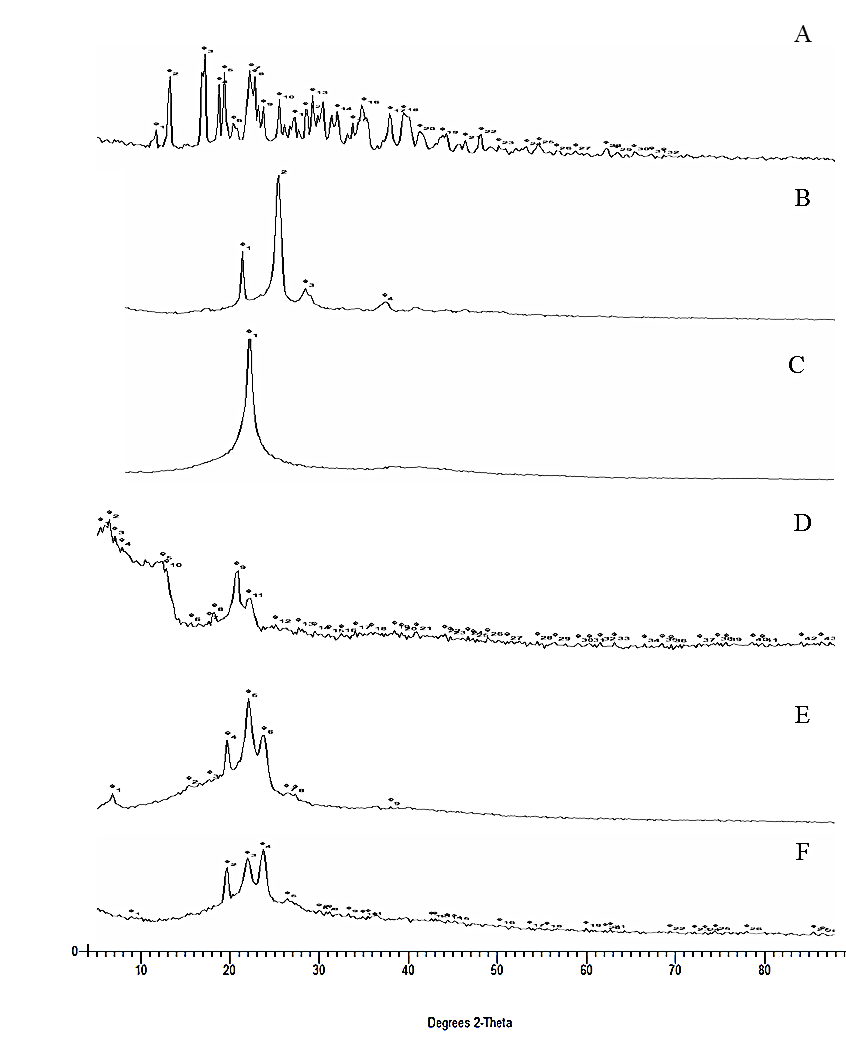


# Factorial Optimization of MRP-Spanlastics Formulation:

A full three-level response surface factorial experimental design was carefully constructed and subjected to thorough statistical analysis using Design-Expert® software (version 12, Stat-Ease, Inc., MN, USA). Polynomial analysis was applied to all response variables, revealing that the two-factor interaction model (2FI) best fit the data for particle size and zeta potential, while the linear model was most appropriate for analyzing the polydispersity index (PDI) and encapsulation efficiency (EE%). For drug release, the Mean model provided the optimal fit. The evaluation of the data was based on three critical statistical indicators: adequate precision, adjusted R², and predicted R². Adequate precision, which measures the signal-to-noise ratio, is crucial for model selection, with a ratio above 4 being desirable to guide experimental design exploration (Lima et al. 2011). In this study, all responses demonstrated ratios exceeding this threshold (**Table 4**). Adjusted R² reflects the proportion of variability explained by the model relative to the mean, while predicted R² indicates the model's ability to predict new data variability. Notably, the predicted R² for all responses, except zeta potential, was negative, signifying that the overall mean was a better predictor than the model for these responses (Chauhan and Gupta 2004, Kaushik et al. 2006).

Optimization techniques were employed to identify the most suitable formulation for further studies. Simultaneously achieving the desired outcomes for all responses is often challenging, as optimizing one response may negatively impact others. The software utilizes a desirability function, which mathematically integrates all responses into a single value, to predict the ideal factor levels (Bendas and Abdelbary 2014, Qelliny et al. 2019). The goal was to minimize particle size, PDI, and cumulative drug release at 6 hours while maximizing zeta potential and encapsulation efficiency. The **F5 batch**, comprising a span-to-edge activator molar ratio of 1:4, a drug concentration of 5 mg/mL, and Brij 35 as the edge activator, exhibited the highest desirability value of 0.379 (**Table 5**). Consequently, this formulation was selected for further in-vivo studies.

**Independent variables of factorial design for MRP spanlastic formulations.**

| **Responses** | **Y1: Particle size (nm)** | **Y2: PDI** | **Y3: Zeta potential (mV)** | | **Y4: EE (%)** | | **Y5: Release at 24 hr** |
| --- | --- | --- | --- | --- | --- | --- | --- |
| Analysis Type | Polynomial | Polynomial | Polynomial | | Polynomial | | Polynomial |
| Fitted Model | 2FI | Linear | 2FI | | Linear | | Mean |
| Maximum Value | 4799 | 1 | 0 | | 69 | | 50 |
| Minimum Value | 0 | 0 | -21.5 | | 0 | | 0 |
| Ratio | - | - | - | | - | | - |
| Transformation | None | None | None | | None | | None |
| Adequate Precision | NA | 4.71 | 10.23 | | 5.28 | | NA |
| Adjusted R^2^ | 0.000 | 0.2104 | 0.6571 | | 0.2577 | | 0.0000 |
| Predicted R^2^ | -0.1211 | -0.0707 | 0.4204 | | -0.0801 | | -0.1211 |
| Significant Factors | Intercept | Intercept | Intercept | | Intercept | | Intercept |
| **Optimized Formula (F5)** | | | | | | | |
| **Observed Responses** | 462 | 0.5 | -7.4 | | 69 | | 20 |
| **Predicted Responses** | 1263 | 0.55 | -5.17 | | 29.3 | | 5.16 |
| **Desirability Value** | 0.379 |  | |  | |  |  |

**
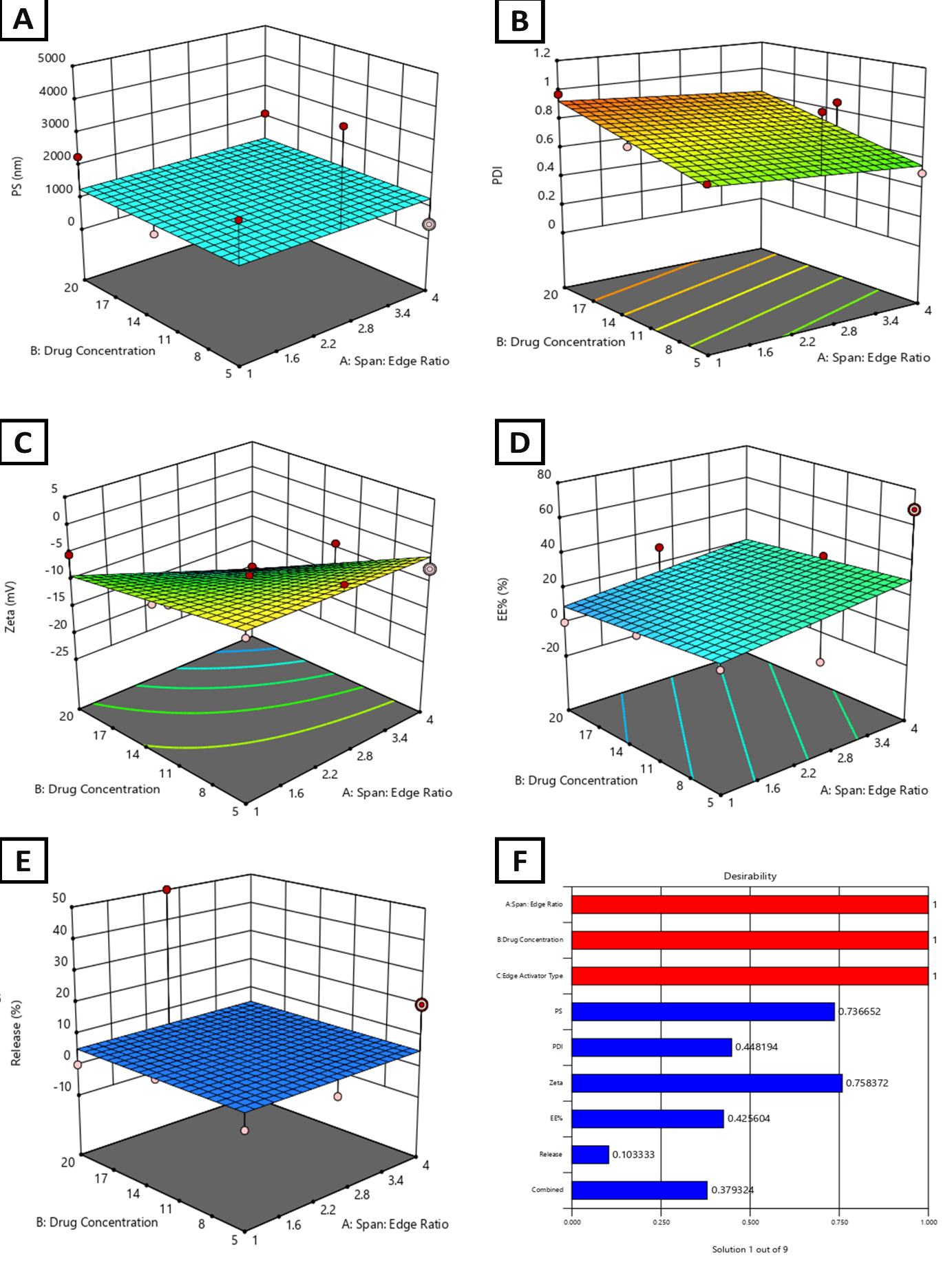
**

Plot in 3- dimensions illustrating the effect of independent factors on particle size (A), PDI (B), zeta potential (C), EE% (D) and release (%) (E). The desirability value for each variable and overall desirability (F).

**References:**

Bendas, E. R. and Abdelbary, A. A. (2014) Instantaneous enteric nano-encapsulation of omeprazole: pharmaceutical and pharmacological evaluation. *Int J Pharm,* *468*(1-2), pp. 97-104.

Chauhan, B. and Gupta, R. (2004) Application of statistical experimental design for optimization of alkaline protease production from Bacillus sp. RGR-14. *Process Biochemistry - PROCESS BIOCHEM,* *39*, pp. 2115-2122.

Cielecka-Piontek, J., Paczkowska, M., Lewandowska, K., Barszcz, B., Zalewski, P. and Garbacki, P. J. C. C. J. (2013) Solid-state stability study of meropenem–solutions based on spectrophotometric analysis. *7*, pp. 1-9.

Farmoudeh, A., Akbari, J., Saeedi, M., Ghasemi, M., Neda, A. and Nokhodchi, A. (2020) Methylene blue-loaded niosome: preparation, physicochemical characterization, and in vivo wound healing assessment. *Drug Deliv Transl Res,* *10*.

Kaushik, R., Saran, S., Isar, J. and Saxena, R. K. (2006) Statistical optimization of medium components and growth conditions by response surface methodology to enhance lipase production by Aspergillus carneus. *Journal of Molecular Catalysis B: Enzymatic,* *40*, pp. 121-126.

Lima, L., Araujo, M., Percio, S., Migliorini, D. and Garcia, J. (2011) Adsorption modeling of Cr, Cd and Cu on activated carbon of different origins by using fractional factorial design. *Chemical Engineering Journal - CHEM ENG J,* *166*, pp. 881-889.

Maghsoodi, M., Montazam, S. H., Rezvantalab, H. and Jelvehgari, M. (2020) Response Surface Methodology for Optimization of Process Variables of Atorvastatin Suspension Preparation by Microprecipitation Method Using Desirability Function. *Pharmaceutical Sciences,* *26*, pp. 61-74.

Mangal, S., Park, H., Zeng, L., Yu, H. H., Lin, Y. W., Velkov, T., Denman, J. A., Zemlyanov, D., Li, J. and Zhou, Q. T. (2018) Composite particle formulations of colistin and meropenem with improved in-vitro bacterial killing and aerosolization for inhalation. *Int J Pharm,* *548*(1), pp. 443-453.

Mhango, E. K. G., Kalhapure, R. S., Jadhav, M., Sonawane, S. J., Mocktar, C., Vepuri, S., Soliman, M. and Govender, T. (2017) Preparation and Optimization of Meropenem-Loaded Solid Lipid Nanoparticles: In Vitro Evaluation and Molecular Modeling. *AAPS PharmSciTech,* *18*(6), pp. 2011-2025.

Qelliny, M. R., Aly, U. F., Elgarhy, O. H. and Khaled, K. A. (2019) Budesonide-Loaded Eudragit S 100 Nanocapsules for the Treatment of Acetic Acid-Induced Colitis in Animal Model. *AAPS PharmSciTech,* *20*(6), pp. 237.
